# Insecure Attachment Styles and Unbalanced Family Functioning as Risk Factors of Problematic Smartphone Use in Spanish Young Adults: A Relative Weight Analysis

**DOI:** 10.3390/ejihpe11030075

**Published:** 2021-09-04

**Authors:** Sonia Mangialavori, Claudia Russo, Maria Veronica Jimeno, Jorge Javier Ricarte, Giulio D’Urso, Daniela Barni, Marco Cacioppo

**Affiliations:** 1 Department of Human Sciences, LUMSA University, Piazza delle Vaschette 101, 00193 Rome, Italy; c.russo@lumsa.it (C.R.); m.cacioppo@lumsa.it (M.C.); 2 Department of Psychology, School of Medicine, University of Castilla La Mancha, 02071 Albacete, Spain; veronica.jimeno@uclm.es (M.V.J.); jorgejavier.ricarte@uclm.es (J.J.R.); 3 School of Education, University College Dublin, Belfield, Dublin 4, Ireland; giulio.durso@ucd.ie; 4 Department of Human and Social Sciences, University of Bergamo, 24129 Bergamo, Italy; daniela.barni@unibg.it

**Keywords:** problematic smartphone use, family functioning, attachment styles, young adults, relative weight analysis

## Abstract

Young adulthood is the life stage during which people are more prone to develop problematic smartphone use (PSU). Only one study investigated the relationship among attachment styles, family functioning, and PSU, but thus far, no research has shown the relative importance that such dimensions may have on PSU. The main aim of this study was to analyze to what extent insecure attachment styles and unbalanced family functioning are related to PSU, investigating the specific weight of each dimension in a sample of young adults (N = 301; 82.7% females; M_age_ = 22.89; SD = 3.02). Participants completed a self-report questionnaire, including the Relationship Questionnaire, the Family Adaptability and Cohesion Evaluation Scale IV, and the Smartphone Addiction Scale. The regression and relative weight analyses results showed that preoccupied attachment style and disengaged, chaotic, and enmeshed family functioning were positively related to PSU. Implications for future research and interventions were discussed.

## 1. Introduction

Smartphones allow extensive internet use, making a huge variety of apps available, including texting, gaming, and social networking [1]. Today, smartphones represent approximately 90% of used devices in Spain [2], and they are an essential part of daily activities used to transform individuals’ lives. This transformation has caused an increase in concerns about problematic behaviors linked to their overuse, strongly associated with the growing number of applications or “apps” offered by smartphones [3]. Adverse consequences of compulsive and excessive smartphone use that is also labeled problematic smartphone use (PSU) [4], include elevated risks of developing psychopathologies such as attention deficit, anxiety, and depression [4,5,6]. According to several researchers, PSU can be conceptualized as excessive attention and concern for smartphones and their related apps, a loss of control over the time spent on a smartphone, and a sense of discomfort where access to a smartphone is limited or impossible [7,8]. 

Despite controversy in the scientific literature about PSU conceptualization as an addictive disorder, Billieux and colleagues [7] argued a lack of research in this field in documenting significant behavioral and neurobiological similarities with more widely recognized substance addictions. Indeed, few studies have documented the presence of loss of control (i.e., trouble consciously limiting one’s smartphone use), tolerance (i.e., increasing smartphone use to achieve satisfaction), and withdrawal (i.e., negative symptoms that occur after smartphone use discontinuation) [7]. Moreover, the lack of enough research on the topic does not allow scholars to reach a univocal definition of the phenomenon. It is currently unclear whether “problematic” should be defined by the quantity of use, patterns of use, or the negative consequences of usage. According to DSM 5 [9], in order to be assessed as an addiction, PSU should include all the three abovementioned criteria (i.e., loss of control, tolerance, and withdrawal), as well as other additional criteria (e.g., “recurrent use in situations in which it is physically hazardous” or “continued use despite having persistent or recurrent social or interpersonal problems caused by or exacerbated by use”).

During the last decade, several studies involving groups of adolescents or young adults tried to identify individual risk factors associated with PSU. Certain risk factors were related to gender, age, anxiety, and personality traits. Specifically, previous findings showed that females were more likely to develop PSU than males [10,11], as well as young age being more related to the overuse of smartphones [11,12,13]. As far as anxiety and personality traits were concerned, anxiety [1], impulsivity [12], extroversion, and neuroticism [14] were all positively associated with PSU. Other individual risk factors associated with PSU were emotional dysregulation [15], self-dysregulation [16], low self-control [17], and behavioral inhibition [18].

Only recently, with the aim to better understand PSU and its risk factors, research has extended the focus from individual factors to relational and family ones. For example, two studies [19,20] explored the relationship between attachment styles and PSU in a group of college students, discovering the significant positive link between insecure attachment styles and PSU. Moreover, a preliminary study showed that during adolescence, an insecure attachment style was associated with an overinterpretation of other’s mental states, and, in turn, it was linked with feedback-seeking on social media [21]. Finally, another recent study focused on the association between PSU and family relationship quality in Lebanese undergraduate students [1]. Its results showed that students who reported several problems related to family relations, such as lack of family communication and family cohesion (i.e., emotional closeness to family members), reported high levels of PSU.

However, the above-mentioned studies investigated the role of attachment styles or family dimensions separately, despite a theoretical and empirical interdependence between them. First, the construct of attachment styles and family functioning are, to an extent, overlapped. For example, Minuchin [22] categorized familial relationships based on their levels of adaptability (secure attachment), enmeshment (ambivalent attachment), or disengagement (avoidant attachment). Most importantly, attachment styles can influence family relationship quality [23,24], and family functioning can influence attachment styles [25,26]. For example, having an insecure family base does not enable family members to show their authentic feelings freely (e.g., express anger or have disagreements) because their family representation does not lead them to the awareness of unconditional love [23]. In this regard, Harvey and Byrd [27] showed that adolescents and young adults characterized by high levels of insecure attachment were concurrently characterized by negative perceptions of their family relationships, reporting high levels of familial conflict and control.

The joint contribution of attachment and family functioning to the onset of dysfunctional behaviors is well known [28,29,30,31]. Recognizing that attachment relationships do not occur in isolation but rather within the larger family system, several authors argued that the accurate study of attachment should include family processes such as emotional bonding that family members have toward one another, especially for understanding risky behaviors such as suicide attempts, substance use and problematic internet use [24,32,33,34,35]. Looking at family functioning and attachment styles together is essential, not only because they share a common theoretical background, but mainly because of the potential therapeutic implications. Specifically, these models conceptualize the problem as the result of attachment insecurities, and they view the problem maintenance as the result of a cybernetically controlled system. In addition, the treatment is focused on changing the specific interactions that maintain the problem with a focus on improving attachment issues (such as emotional nurturance) underlying the problematic behavior.

In this regard, Cacioppo et al. [32], involving a non-clinical sample of Italian adolescents, examined these variables (i.e., attachment styles and family functioning) as predictors of the problematic internet use (PIU). Results suggested that a greater anxious-preoccupied attachment is associated with a greater risk of PIU. In contrast, family affective involvement as well as clearly and equitably assigned tasks to family members were negatively associated with PIU. Meanwhile, Şenormancı et al. [36] investigated these same variables in a clinic sample of adolescents with Internet addiction disorder (IAD). Results showed that adolescents with IAD scored higher on attachment anxiety scale and perceived their family functioning as more negative (i.e., weak emotional bonds among family members and family’s inability to change their power structure in response to situational stress) than the control group. Although PSU and PIU/IAD can be included in the macro-category of cybertechnology problematic behaviors [10], they only partially overlap. Contrary to what happened several years ago, when Internet use was possible only by computers or laptops, smartphones have destroyed the space-time wall, allowing uninterrupted access to the web and apps. At the beginning, many prominent communication channels were exclusive to smartphones and could not be accessed via desktop computers (e.g., Snapchat, Telegram, and Whatsapp). The messenger service WhatsApp is a good example of a successful application dominating smartphone usage in everyday life [37]. This application has recently become available on desktop computers, but its use by smartphones remains more popular. Empirical studies have shown that PIU’s risk factors differ from the risk factors related to PSU both in adolescence and young adulthood [38]. For example, in their study, Choi et al. [38] found that higher PIU scores were associated with higher levels of PSU and vice versa, but they identified the female gender as a risk factor for PSU, whereas males were more at risk for PIU. This would depend on females and males using the Internet for different purposes. In most cases, females prefer to use the Internet to chat and use messenger systems, while males are likely to use the Internet for gaming online [20]. Thus, females can engage in their favorite activities using smartphones, whereas males cannot use smartphones for gaming online because a smartphone’s capability and graphic power are generally lower than those of computers [38]. Other authors argued that females can engage in gaming online, but they continue to prefer their smartphones and related gaming apps (e.g., Candy Crush Saga) rather than personal computers [39]. However, two recent studies [3,40] involving Spanish participants showed that there were no gender differences in general PSU, but for specific patterns of its use such as chatting, females were more prone to use their devices for communication purposes. De Sola et al. [3] also reported significant age differences, showing that problematic users were mainly young adults. Young adulthood is a life stage still characterized by identity explorations, as well as by high levels of uncertainty regarding the future, especially in Mediterranean countries such as Spain [41]. Considering these statements, previous evidence suggested that young adulthood is a critical period for the development of addictions [42], especially in the case of online-related addictions [43]. Csibi et al. [44], analyzing PSU across different age groups, found that young adults (aged 20 to 34) were at the highest risk for smartphone-related addictive behavior, with higher scores in tolerance, withdrawal symptoms, and relapse subscales than other age groups.

Only recently, Jimeno et al. [45] analyzed the relationship among attachment styles, perceived family functioning, and PSU in a group of young Spanish adults. Results showed that the cohesion and enmeshed family functioning were the best predictors of PSU. At the same time, preoccupied attachment was the only one that also showed indirect effects on problematic smartphone use through the variable of enmeshed family functioning [45]. However, despite these promising findings, the authors did not consider the high statistical collinearity among predictors and between them and mediators. Multicollinearity may produce confusing results, leading to wrong interpretations. Moreover, highlighted above, there is also a theoretical interdependence among these variables [23,24,25,26]. Thus, it may be hazardous to determine which comes first. As such, it may be relevant to analyze the relative importance of each dimension (i.e., attachment styles and family functioning) in explaining PSU. 

### The Present Study

Considering this background, the main aim of this study was to determine the relative importance of insecure attachment styles (i.e., fearful, preoccupied, and dismissive styles) and dimensions of unhealthy family functioning (i.e., rigidity, disengagement, enmeshment, and chaos) in explaining PSU in a sample of young Spanish adults. 

In order to identify risk factors of PSU, we considered the three adults’ insecure attachment styles proposed by Bartholomew and Horowitz [46]. Specifically, an anxious-preoccupied attachment style is demonstrated by those who possess a negative view of self and a positive view of others. A dismissive–avoidant attachment style is demonstrated by those possessing a positive view of self and a negative view of others, and finally a fearful–avoidant attachment style is demonstrated by those possessing an unstable fluctuating or confused view of self and others [47].

Moreover, risk factors related to family functioning were conceptualized in accordance with the Circumplex Model [48]. This model suggests that families characterized by too high or too low levels of cohesion (i.e., emotional bonding among family members) and of flexibility (i.e., amount of change in family’s leadership, role relationships and relationship rules) tend to be more dysfunctional and unable to face family challenges. There are four levels of cohesion, ranging from disengaged (very low) to separated (low to moderate) to connected (moderate to high) to enmeshed (very high). It is theorized that extreme levels of cohesion (disengaged and enmeshed) make for unhealthy family functioning, and they are generally seen as problematic for relationships over the long term. The four levels of flexibility range from rigid (very low) to structured (low to moderate) to flexible (moderate to high) to chaotic (very high). As with cohesion, it is hypothesized that extreme levels of flexibility (rigid and chaotic) are more critical for the family functioning over the longer term.

Based on the available data in the literature [32,36,45], we expect that insecure attachment, especially the preoccupied one, and unhealthy family functioning increase the risk for PSU in our participants.

## 2. Materials and Methods

### 2.1. Participants and Procedure

Participants were 301 young adults (82.7% females), aged between 19 and 35 (M_age_ = 22.89; SD = 3.02). All the participants come from the center of Spain. Most participants were recruited during the university courses they attended, and data were collected by online questionnaire. After explaining the general aim of the study, a research member provided the link of the survey to the students who agreed to participate. All participants gave their consent for their research participation before completing the questionnaire. The questionnaire took approximately 20 min. 

This study was conducted according to the European law of privacy and informed consent (GDPR 2016/679) and according to the ethical guidelines of the Spanish Psychological Association (SPAIN) and of the *Declaration of Helsinki*. It was approved by the Ethical Committee of the University of Castilla La Mancha, Albacete, Spain (N° 2018/10/105).

### 2.2. Measures

Insecure Attachment Styles. In order to assess insecure attachment styles, we used the Relationship Questionnaire (RQ) [46,49]. The RQ consists of four descriptions of the different types of adult attachment patterns (i.e., secure, fearful, preoccupied, and dismissing) and respondents were asked to report their own similarity to each prototype (from 1 = does not describe me, to 7 = describes me very accurately). For the present study, only the items related to the insecure attachment (i.e., fearful, preoccupied, and dismissing) were used. An example of item is “I am comfortable without close emotional relationships. It is very important to me to feel independent and self-sufficient, and I prefer not to depend on others or have others depend on me” (dismissing attachment). 

Unbalanced Family Functioning. The four unbalanced subscales of the Spanish version of the Family Adaptability and Cohesion Evaluation Scale (FACES IV) [48,49,50] were used. Specifically, the four negative subscales (4 items for each subscale) describe problematic family functioning in terms of disengagement, enmeshment, rigidity, and chaos. Participants were asked to think about their family of origin and to rate how much they were agreed with each item using a 5-point Likert scale (1 = strongly disagree, 5 = strongly agree). An example of item is: “It is unclear who is responsible for things (chores, activities) in our family” (chaotic). The Cronbach’s alpha coefficients ranged from a minimum of 0.64 (enmeshed) to a maximum of 0.80 (rigidity). 

Problematic Smartphone Use (PSU). The short version of the Smartphone Addiction Scale [40,51] was used. The scale consists of 10 items ranging from 1 (strongly disagree) to 6 (strongly agree) and evaluates the problematic use of smartphones. The scale is considered an appropriate tool for diagnosing smartphone addiction, as well as for identifying a problematic use of it. An example of an item is: “Missing planned work due to smartphone use” (α = 0.86). 

### 2.3. Data Analysis

The SPSS-20 software for Windows was used. First, we described the study variables in terms of mean, standard deviation, and range. For descriptive purposes, we also performed t-test of study variables to assess gender differences. Associations between the variables were measured by bivariate Pearson correlation. Additionally, we conducted a hierarchical multiple regression (HMR) in which predictors were entered in three steps. Specifically, young adults’ gender (1 = males; 2 = females), age (Step 1), insecure attachment styles (Step 2), and unbalanced family functioning (Step 3) were entered as predictors of PSU. In this way, we estimated the overall R^2^ and determined the statistical significance of individual regression coefficients. However, when predictors are correlated both theoretically and, above all, statistically—as likely in the case of attachment styles and family functioning dimensions—HMR does not adequately divide the explained variance of the criterion among the predictors [52]. To face multicollinearity, we supplemented HMR with relative weight analysis (RWA), which provides information about the impact of a predictor relative to the others in the model, considering both its unique contribution on the criterion variable and its contribution when combined with the other predictors. The important weights provided by the analysis is scaled in the metric of relative effect size by dividing the relative weights by the total R^2^ and multiplying the values by 100. The rescaled weight is interpreted as the percentage of predicted criterion variance attributed to each predictor [53]. 

## 3. Results

Descriptive statistics of the study variables are reported in Table 1. 

All variables showed a normal distribution, as suggested by the skewness and kurtosis indicators, which ranged from −2.00 to +2.00. Among the attachment styles, the highest mean score was recorded for fearful. The highest score recorded among the family functioning dimensions was the enmeshed one. Moreover, the t test analysis for gender showed that there was a difference in mean level for preoccupied attachment style [t(1, 299), −2.461, *p* = 0.014], and for disengaged [t(1, 299), 2.347, *p* = 0.020] and enmeshed [t(1, 299), −2.136, *p* = 0.034] family dimensions. Specifically, for preoccupied attachment style, females showed higher mean levels (M = 2.98, SD = 1.51) than males (M = 3.63, SD = 1.76). For enmeshed family functioning, females reported higher mean levels (M = 10.99, SD = 3.18) than males (M = 9.98, SD = 3.66). On the contrary, for disengaged family dimension, males showed higher mean levels (M = 10.06; SD = 2.41) than females (M = 9.18, SD = 2.46). For all the other study’s variables, t test analysis showed there was not a significant difference: fearful [t(1, 299), 0.218, *p* = 0.827], dismissing [t(1, 299), −1.398, *p* = 0.163], chaotic [t(1, 299), −1.513, *p* = 0.131], rigid [t(1, 299), 0.362, *p* = 0.718], and PSU [t(1, 299), −0.164, *p* = 0.860]. 

Table 2 shows the Pearson correlation coefficients between the study variables.

Strong correlations, including between predictors, emerged. Both attachment styles and family dimensions were intercorrelated (e.g., fearful with dismissing, *p* < 0.01; disengaged with chaotic, *p* < 0.01). Furthermore, PSU was significantly and positively associated with both preoccupied (*p* < 0.01) and dismissing (*p* < 0.05) attachment styles as well as with both disengaged (*p* < 0.01) and enmeshed (*p* < 0.05) family dimensions. 

Table 3 reports the MR and RWA results.

Overall, the regression model, which was statistically significant (*F* (9, 291) = 3.801, *p* < 0.01), accounted for 10.5% of the variability of PSU. Inspection of β coefficients suggested that preoccupied attachment (*β* = 0.14, *p* = 0.022), disengaged (*β* = 0.20, *p* < 0.006) and enmeshed (*β* = 0.18, *p* = 0.003) family dimensions were significantly related to PSU, specifically, the higher the above-mentioned dimensions, the higher PSU.

RWA partly confirmed MR results. It confirmed the importance of preoccupied attachment style (11.2%) and enmeshed family functioning (21.5%), which together, accounted for 32.7% of the explained variance of PSU. Moreover, RWA reinforced the substantial importance of the disengaged dimension (34.8%), and chaotic family functioning gained more relevance in predicting PSU (21.5% of the explained variance).

## 4. Discussion

Currently, PSU is an increasing phenomenon that mainly affects young adults [3]. People, especially young people, tend to spend a long time on smartphone for several reasons, for example, chatting or scrolling in social networks. Due to the relevance of this problematic behavior, the main aim of this study was to assess the relative importance of insecure attachment styles and unhealthy family functioning dimensions in predicting PSU in a sample of young Spanish adults. 

Our results showed that the major risk factors for PSU were family variables, namely: disengaged, chaotic and enmeshed family functioning. These results are consistent with previous studies that underlined how poor family functioning and insecure attachment styles are related to PSU [1,32,45]. Specifically, for young Spanish adults, the lack of proper leadership in the family or role changes are strongly related to smartphone overuse. The inefficient family function is related to individual psychological unbalancing, leading to an undesirable emotional state. In order to avoid this undesired emotional state, the person may rely on a technological tool with interactive features, such as a smartphone [15,30]. In addition, as previous studies showed, for young Spanish adults, an enmeshed family functioning was negatively correlated with their psychological wellbeing [45,54]. The Spanish population is characterized by a strong cultural emphasis on autonomy and individuation, and as such, young adults tend to create distance between themselves and their families [54]. For this reason, an enmeshed family functioning could impair both young adults’ autonomy and self-differentiation, increasing their risk of developing PSU. They can exercise control from smartphones and have greater decision-making autonomy [55]. Conversely, having a disengaged family system characterized by a lack of closeness and relatedness among family members and excessive emphasis on independence is a risk factor for PSU. A family characterized by members who do not have enough time or a desire to address other family members does not represent a positive environment for young adults. It implies that young adults who have experienced their parents as emotionally distant and who are unable to set and maintain roles and relationship rules are more prone to becoming problematic smartphone users. This finding is consistent with previous studies that suggested a link between PSU and family relations [1]. A family environment characterized by high levels of chaos is composed of members that are emotionally distant and unable to set and clearly maintain roles and relationship rules in response to situational and developmental stress.

Our results indicate that the young Spanish adults’ perception of lack of emotional bonding between family origin members and the lack of clear roles and rules may be the major risk factors for developing PSU. As Elhai et al. [56] highlighted, PSU can be conceptualized as a maladaptive coping strategy. As such, people with an unbalanced family functioning may refer to PSU as an emotion compensator to regulate or alleviate negative emotions (e.g., fear, boredom, and sadness), possibly because the device is constantly accessible and may be the first and most obvious object to deviate the negative family’s context. 

Regarding attachment styles and consistent with previous findings [45], our results underlined that insecure attachment, especially the preoccupied one, was related to PSU. People who have a preoccupied style have a negative view of self and a positive view of others, and they are characterized by an excessive dependency on others’ approval in close relationships and by an over-involved, demanding interpersonal style [46]. Several previous studies have demonstrated how an insecure attachment style (especially the preoccupied one) can lead young people to use smartphones as a virtual retreat in order to protect themselves from feelings of loneliness, fears about real interactions, and a sense of ineffectiveness in close relationships. It plays a role in the onset of PSU during the young adulthood [19,45]. Young adults can also conceive the smartphone as a safe base where they can try to interact with other people to develop a better sense of closeness, connectedness, competence, and therefore, self-therapy [19,20].

Concerning gender, consistent with Lopez-Fernandez [40] and De Sola et al. [3], we found that for young Spanish adults being a female was not a risk factor for developing PSU. This finding may be because in Spain, the smartphone is equally used by both female and male young adults [40] and because we explored the self-perception of PSU without considering the use of the different applications such as social media, browsing, and listening to music. De Sola et al. [3] showed that Spanish females are more at risk of PSU than males, only for chatting applications, and they found no gender differences for other types of applications. In addition, we found no age differences among our participants; our sample was mainly composed of young adults with a low average age, and they are usually considered the most at risk of PSU [3,40].

There are methodological limitations in the present study. First, only self-reported measures were used, and the cross-sectional nature of research design cannot allow us a causal interpretation of results. A longitudinal design will be needed to provide a developmental perspective on PSU during a lifespan. Second, participants were selected through a non-probability sampling and were composed mainly by females. The higher prevalence of females in our sample did not exclude a gender bias in our results, and for this reason, we could not compare our regression results based on gender. For this reason, future studies should be more homogeneous about the gender of participants to better explore possible differences between the male and female population in the onset of PSU. Third, we assessed only the self-perception of PSU without considering the different patterns of usage. Future studies should integrate these findings involving questions about the use of different smartphone applications, including for better understanding potential differences in gender, attachment styles, and family functioning. 

Despite these limitations and to the best of our knowledge, this study is the first one to treat the theoretical and statistical interdependence among young adults’ attachment styles and family functioning dimensions in predicting PSU. In doing this, we used a new analytic strategy (i.e., RWA) to supplement MR. RWA extends beyond this to understand the impact of a particular predictor more fully within the context of other predictors [53]. Moreover, in the case of highly correlated predictors, as in the case of attachment styles and family dimensions, RWA contributes to making regression results more meaningful and interpretable. 

In summary, this study underlined the importance for clinicians to consider not only individual dimensions (such as attachment styles, gender, and age), but also familiar ones, in order to adopt a comprehensive and integrative view for the implementation of preventive treatments against problematic cyber-behaviors. The present findings suggest that it can be useful to assess the role of a preoccupied attachment style and a dysfunctional family functioning and eventually treat these problems for those who display high PSU levels. This may help young adults better understand the origin of their problematic behaviors and learn how to cope with their psychological difficulties, which may reduce their risk of compulsively using a smartphone as a dysfunctional strategy to escape from their internal and familiar problems.

For example, clinical interventions can promote an understanding that individuals who struggle with the perceived unreliability of close others (such as their own family members) may use their smartphones as an alternative source of security, confidence, and anxiety reduction. Thus, for young adults, their PSU may be read as a protective and compensatory function of their dysfunctional family environment that provides a source of confidence or anxiety reduction in new situations [19,20]. Being aware of using smartphones as a compensatory and protective strategy to relieve the pain caused by the perception of the unavailability of close others can help young adults implement different and more adaptive strategies, such as identifying and communicating their mental states to significant others. For this reason, interventions targeting emotional literacy and mentalization processes may be particularly beneficial in buffering their PSU.

## Figures and Tables

**Table 1:** Descriptive statistics of the study variables.

	M	SD	Range	Skewness	Kurtosis
Insecure Attachment Styles					
Fearful	4.49	1.69	1–7	−0.97	−0.23
Preoccupied	3.51	1.73	1–7	−1.01	0.35
Dismissing	3.72	1.80	1–7	−1.27	0.12
Unbalanced Family Functioning					
Disengaged	9.33	2.72	3–14	0.31	0.64
Chaotic	6.81	2.81	3–15	−0.14	0.69
Enmeshed	10.82	3.12	4–19	−0.31	0.12
Rigid	9.82	3.58	4–20	−0.35	0.43
Problematic Smartphone Use	26.23	9.07	10–56	0.25	0.70

**Table 2:** Bivariate correlations matrix of the study variables.

	1	2	3	4	5	6	7	8
Insecure Attachment Styles								
1. Fearful	-	0.02	0.18 **	0.02	0.06	−0.11	−0.01	−0.02
2. Preoccupied		-	0.36 **	0.23 **	0.20 **	0.18 **	0.13 *	0.17 **
3. Dismissing			-	0.23 **	0.18 **	0.06	0.10	0.12 *
Unbalanced Family Functioning								
4. Disengaged				-	0.55 **	−0.25 **	0.16 **	0.22 **
5. Chaotic					-	−0.14 *	0.01	0.20 **
6 Enmeshed						-	0.15 **	0.13 *
7. Rigid							-	0.06
8. Problematic Smartphone Use								-

Note: ** *p* < 0.01, * *p* < 0.05.

**Table 3:** Hierarchical multiple regression (HMR) and relative weights analysis (RWA) results.

	MR				RWA	
	*β*	t	*p*	VIF	Raw Importance	Rescaled Importance
Gender	0.00	0.08	0.935	1.0	0.002	1.8%
Age	−0.02	−0.42	0.678	1.0	0.000	0.4%
Insecure attachment styles						
Fearful	−0.04	−0.73	0.466	1.1	0.001	0.8%
Preoccupied	0.14	2.30	0.022	1.1	0.012	11.2%
Dismissing	0.08	1.28	0.201	1.2	0.006	5.3%
Family Functioning						
Disengaged	0.20	2.79	0.006	1.3	0.037	34.8%
Chaotic	0.11	1.62	0.105	1.2	0.025	23.3%
Enmeshed	0.18	2.99	0.003	1.1	0.023	21.5%
Rigid	−0.01	−0.24	0.811	1.1	0.001	0.8%
R^2^					10.5	100%

## Data Availability

The data presented in this study are available on request from the corresponding author.
